# Treatment of Cardiac Rhabdomyomas with mTOR Inhibitors in Children with Tuberous Sclerosis Complex—A Systematic Review

**DOI:** 10.3390/ijerph18094907

**Published:** 2021-05-05

**Authors:** Monika Sugalska, Anna Tomik, Sergiusz Jóźwiak, Bożena Werner

**Affiliations:** 1 Department of Pediatric Neurology, Medical University of Warsaw, 02-091 Warsaw, Poland; mslowinska@wum.edu.pl; 2 Department of Pediatric Cardiology, Medical University of Warsaw, 02-091 Warsaw, Poland; anna.m.tomik@gmail.com (A.T.); bozena.werner@wum.edu.pl (B.W.)

**Keywords:** tuberous sclerosis complex, everolimus, sirolimus, mTOR inhibitor, cardiac rhabdomyoma, heart, tumor, children

## Abstract

Background: Cardiac rhabdomyomas (CRs) are the earliest sign of tuberous sclerosis complex (TSC). Most of them spontaneously regress after birth. However, multiple and/or large tumors may result in heart failure or cardiac arrhythmia. Recently, the attempts to treat CRs with mTOR inhibitors (mTORi) have been undertaken. We reviewed the current data regarding the effectiveness and safety of mTORi in the treatment of CRs in children with TSC. Methods: The review was conducted according to the PRISMA guidelines. Medline, Embase, Cochrane library, and ClinicalTrial.gov databases were searched for original, full-text articles reporting the use of mTORi (everolimus or sirolimus) in the treatment of CRs in children with TSC. Results: Thirty articles describing 41 patients were identified (mostly case reports, no randomized or large cohort studies). Thirty-three children (80.5%) had symptomatic CRs and mTORi therapy resulted in clinical improvement in 30 of them (90.9%). CRs size reduction was reported in 95.1%. Some CRs regrew after mTORi withdrawal but usually without clinical symptoms recurrence. The observed side effects were mostly mild. Conclusions: mTORi may be considered as a temporary and safe treatment for symptomatic CRs in children with TSC, especially in high-risk or inoperable tumors. However, high-quality, randomized trials are still lacking.

## 1. Introduction

Primary cardiac tumors are rare. The prevalences in the pediatric population vary from 0.0017 to 0.28 in the autopsy series [1]. In children, only about 10% of cardiac tumors are malignant [1]. Cardiac rhabdomyomas (CRs) are the most common cardiac tumors in children [1]. CRs may occur as isolated lesions or be associated with tuberous sclerosis complex (TSC). TSC is a multiorgan genetic disease with an incidence rate of approximately 1:6000–1:13,000 live births [2]. The disease is caused by the mutation of *TSC1* or *TSC2* resulting in the overactivity of the mechanistic target of rapamycin (mTOR) pathway leading to the development of multiple, mostly benign, tumors in different organs, including heart [3]. Early-onset and drug-resistant epilepsy, intellectual disability, and autism spectrum disorder are also common manifestations of TSC [3].

Although both single and multiple CRs are included as a major diagnostic criterion of TSC [4], the likelihood of TSC diagnosis varies depending on the number of CRs. Multiple CRs are currently considered as the earliest clinical biomarker of TSC and are associated with 95% risk of TSC diagnosis [5,6,7]. Single CR, depending on the report, is associated with TSC in 23–73% of cases [5,6,7].

CRs are the earliest sign of TSC and may be detected during pregnancy [8]. Unlike other TSC symptoms, most CRs spontaneously regress with age [9,10]. Therefore, they are usually detected in neonates and infants—they are seen in 66–83% of patients below 2 years of age [9,10]. CRs may also re-grow or occur de novo in adolescence, especially in girls [9].

Most of the CRs are asymptomatic and do not require treatment [9]. However, they can also be the cause of clinical symptoms, including both mild, as a heart murmur, and more severe, e.g., cardiac arrhythmia and heart failure requiring therapeutic intervention [9].

Until recently, surgery or symptomatic treatment with anti-arrhythmic drugs were the only therapeutic options for symptomatic CRs. Nevertheless, surgical resection is associated with significant morbidity and mortality and may be difficult to conduct in some cases, especially when tumors are giant or multiple and in preterm or low birth-weight neonates. In recent years, the better understanding of the role of the overactivity of the mTOR pathway in the pathophysiology of TSC resulted in the development of new therapeutic options such as the use of mTOR inhibitors (mTORi) [11].

Everolimus and sirolimus are mTORi which suppress the activity of the mTOR pathway and may alleviate TSC manifestations. Within the last decade, these drugs have been more and more widely tested and used in the treatment of various TSC symptoms [11]. Clinical double-blind placebo-controlled trials already proved the effectiveness of mTORi in the treatment of brain and kidney tumors (subependymal giant cell astrocytoma (SEGA), angiomyolipoma (AML)), and epilepsy [12,13,14]. mTORi have been also proved to be effective in the therapy of other TSC manifestations, e.g., lymphangioleiomyomatosis or skin lesions [13,15,16,17,18,19]. As dysregulation of the mTOR pathway is also present in CRs, mTORi may be also potentially used in the therapy of CRs in patients with TSC [20]. However, to date, there were no published results of randomized trials nor guidelines regarding the treatment of CRs with mTORi in children with TSC.

The aim of this article is a revision of current data regarding the effectiveness and safety of mTORi in the treatment of CRs in children with TSC to provide summarized and systematic information about this recent possibility of pharmacological intervention that may be used in the clinical practice.

## 2. Materials and Methods

Our review was conducted according to the Preferred Reporting Items for Systematic Reviews and Meta-Analyses (PRISMA) guidelines [21].

### 2.1. Eligibility Criteria

We implemented the following PICOS criteria:

The a priori hypothesis was that the use of mTORi reduces a CRs size and alleviates clinical manifestations of symptomatic CRs.

(a) P (patients)—children (0–18 years) with TSC and CRs
(b) I (intervention)—treatment with mTORi (everolimus or sirolimus)
(c) C (comparator)—At the beginning, we searched for studies comparing the treatment of CRs with mTORi to surgical intervention or non-intervention. We only found one small retrospective study with a historical control group. Therefore, we had modified the criteria and decided to resign from the comparator criterion.
(d) O (outcome)—size/volume of CRs (if possible to determine), influence on the clinical symptoms caused by CRs, and assessment of side effects associated with mTORi.
(e) S (study design)—Only full-text, original studies disregarding of study type. Only studies published in English or Polish.

We included only full-text journal articles including pediatric patients (aged 0–18 years) with TSC and CRs who were treated with mTORi (everolimus or sirolimus). Only studies published in English or Polish were included. No publication date, type or publication status restrictions were imposed.

Articles that did not fulfill the inclusion criteria were excluded from the analysis. Non-full-text articles (e.g., conferences’ abstracts) were not included.

### 2.2. Information Sources

Studies were identified by searching electronic databases: MEDLINE, Embase, and Cochrane Library. Additional reports were identified through the search of references’ lists of included reports and review articles. Additionally, ClinicalTrials.gov was also searched for any past or current trials with mTORi for CRs in children with TSC. The first search was done on the 12th of December 2020 and the last on the 28th of December 2020. Databases were searched by one author (MS).

### 2.3. Search Strategy

We applied the following search phrase: (“tuberous sclerosis”) AND (children OR pediatric OR infants OR neonate) AND (cardiac OR rhabdomyoma OR “heart tumor”) AND (“mTOR inhibitor” OR sirolimus OR everolimus OR rapamycin). Using ClinicalTrials.gov as a search strategy, we only use the disease name “tuberous sclerosis complex”.

### 2.4. Study Selection

Eligibility assessment was performed in the three-phase protocol: (1) title, (2) abstract, and (3) full-text analysis. The first phase was done by one author (MS). The assessments within the second and the third phase were performed by 2 reviewers (MS and AT). The results were compared between the reviewers. Any disagreement was resolved by the discussion and consensus.

### 2.5. Data Collection Process and Data Items

Two reviewers (MS and AT) extracted data from selected articles. The differences between the reviewers were resolved by the discussion and consensus.

To decrease the risk of data duplication, at the end of data collection, we juxtaposed and compared authors’ names and patient’s characteristics. If there was a suspicion of data duplication, issued articles were once again analyzed and compared by MS.

The following information was extracted from each included article: title, author’s name, year of publication, sample size and characteristics, type of intervention, main results considering the effect of mTORi on the CRs’ size and clinical symptoms, side effects of the treatment, and CRs characteristic after mTORi discontinuation.

### 2.6. Assessing the Risk of Bias in Individual Studies

To assess the risk of bias, we had planned to use the Cochrane risk of bias tool for randomized trials and the Newcastle-Ottawa Scale (NOS) for nonrandomized studies. The Cochrane risk of bias tool was not applied as during the search we did not find any randomized trials fulfilling the inclusion criteria.

NOS is a scale in which the study is scored by stars (from 0–9) based on the following criteria: (1) Selection (maximum of 4 stars) including (a) representativeness of the exposed cohort, (b) selection of the non-exposed cohort, (c) ascertainment of exposure, d) whether the outcome of interest was present at the beginning of the study; (2) Comparability of the cohorts on the basis of study design and analysis methodology (maximum of 2 stars); (3) Outcome (maximum of 3 stars) including (a) outcome assessment, (b) follow-up duration, (c) adequacy of follow up cohort [22]. Studies scored with 9–7, 6–4, and 3–0 stars were defined as low, moderate, and high risk of bias, respectively.

The assessment was independently performed by MS and AT. The differences between the reviewers were resolved by the discussion and consensus.

### 2.7. Summary Measures

The primary outcomes measures for this review were: the difference in CRs size before and after the treatment, the effect on clinical symptoms of CRs, and occurred side effects associated with the intervention.

Due to significant differences in the reporting of CRs size between the studies (reporting of 1 vs. 2 vs. 3 dimensions) we summed the available dimensions of the largest or symptomatic CRs in particular cases and calculated the percentage change of summed dimensions. In some articles, the size of CRs after mTORi treatment was not reported. However, the authors reported the percentage of reduction—we included that data in the analysis.

### 2.8. Data Analysis

Data are expressed as median and range or mean and standard deviation, and count with percentages. Due to significant differences in data reporting and the lack of randomized trials or studies with control groups, the comparative analysis of everolimus and sirolimus as well as meta-analysis were not performed due to the high risk of bias.

## 3. Results

### 3.1. Study Selection and Available Literature

**Figure 1:**
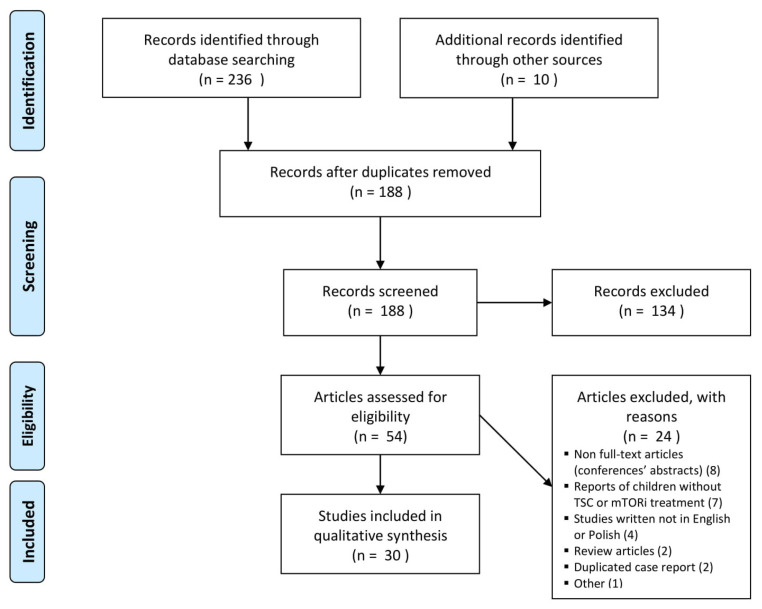
Flow diagram of the study selection.

The search of MEDLINE, Embase, Cochrane Library, and ClinicalTrials.gov provided a total of 236 citations. Additional 10 citations were supplemented from the references’ lists of included or review articles. After adjusting for duplicates 188 remained. Of these, 134 reports were excluded in the first and second phase of the screening as they did not meet inclusion criteria (132 articles reported different than CRs aspects of TSC, and 2 were review articles). Other 24 reports were excluded in the third phase of the searching due to the reasons included in the flow diagram (Figure 1). One study classified as “other” was excluded as it was a notifying article about the planned randomized study of everolimus in CRs treatment [23]. Overall, 30 reports were included in the systematic review [24,25,26,27,28,29,30,31,32,33,34,35,36,37,38,39,40,41,42,43,44,45,46,47,48,49,50,51,52,53]. Three of the included articles partially duplicated the case reports [24,34,53]. We did not double the information. However, to extract the most detailed data we used information contained in all three reports and presented it together as one study (in one line) in Table 1.

### 3.2. Study Characteristics

Studies design: All of the included studies were case reports or case series. Only 1 article reported 4 children treated with mTORi and compared the results with a historical control group of 10 children [24]. Unfortunately, randomized controlled trials, large cohort studies, or case-control studies were not found.

Patients: The included studies involved 41 patients. Only children (patients aged from 0 to 18 years) with TSC and CRs treated with mTORi were included in the analysis.

Intervention: All patients received mTORi, everolimus (28/41 patients (68.3%), 20 out of 30 articles, 66.6%) or sirolimus (13/41 patients (31.7%), 10 out of 30 articles, 33.3%).

Primary and additional outcome: Only one study reported 3 consecutive cases with prospectively defined study protocol [31]. The primary aim of that study was the decrease of CRs size. In other studies, the reported outcomes were the decrease of CRs size and, in most cases, the subsequent alleviation of clinical symptoms.

### 3.3. Risk of Bias within Studies

We only found one small study with a control group [24]. This article was assessed in the Newcastle -Ottawa Scale. The total score was six out of nine stars (two out of four stars in the selection category, one out of two in the comparability, and three out of three in the exposure). Therefore, the article was qualified as a moderate risk of bias.

Other included reports were case series and case reports without control groups. Hence, all of these reports were rated as high risk of bias.

### 3.4. Results of Included Studies

Data extracted from individual studies are presented in Table 1 and Table 2. Among patients included in the analysis there were 23 boys (56%) and 11 girls (26%). In 7 patients (18%) the sex was unknown. Furthermore, 19 children (46.3%) were born in term, while 13 children (31.7%) were born before the term. In nine children (22%) there was no information considering the week of birth. In the majority of patients, CRs were multiple (32 children, 78%). One child had a single CRs (2.5%) and for eight children (19.5%) there was no data available considering the number of CRs. The largest or symptomatic CRs were mostly localized in ventricles and intraventricular septum (28 children, 63.3%). In 11 patients (26.8%), CRs encroached to or were localized in more than one cardiac cavity. In 12 children (29.3%) there was no data available considering CRs localization.

In the majority of patients, the mTORi was introduced in the neonatal period (29/41, 70.7%) or infancy (eight in 41, 19.5%). The youngest child was treated since the second day of life, the oldest since the fifth year of age. Twenty out of thirty articles (66.6%) reported treatment duration that varied from 28 to 390 days (mean 112.6 days, median 70 days). Two patients (4.9%) were still treated with mTORi when the studies were published.

#### 3.4.1. Doses of mTORi

The doses of mTORi significantly differed between studies and were reported in different units. Hence, the direct comparison between the studies was impossible. For 7 patients (17.1%) the information about the mTORi dose was not available. The doses of everolimus were more unified as some authors (six patients, 21.4% in the everolimus group) based on the doses applied in randomized studies of everolimus in other TSC manifestations (EXIST 1 and 2 trials)—that is 4.5 mg/m^2^/week (about 0.1 mg/day) [12,13]. For other patients, the doses of everolimus varied from 0.05 mg to 1 mg and were implemented in different dosing schedules—from daily dosing to two days per week (Table 1). In most of the studies reporting the information about targeted everolimus level, the targeted level corresponded with the EXIST studies that is 5–15 ng/mL (Table 1). In the group treated with sirolimus, the initial dose varied greatly between the studies from 0.25 mg/day to 1 mg/m^2^ twice a day (Table 2). The targeted level of sirolimus ranged from 4 to 20 ng/mL, mostly 5–15 ng/mL (Table 2).

The mTORi blood concentration was measured in different time points or data were unavailable. Therefore, it was difficult to compare the sirolimus and everolimus blood concentration between the patients and correlate it with the dose. However, when the drug was introduced in neonates it sometimes tended to result in blood levels highly above the targeted level, both in sirolimus (5/13, 28.5%) and everolimus (4/28, 14.3%) group, even up to several dozen of ng/mL (the highest reported level was 108 ng/mL for everolimus and 69.7 ng/mL for sirolimus) (Table 1 and Table 2).

#### 3.4.2. Effect of mTORi on CRs Size

Timepoints of the assessment of the effect of mTORi on CRs size and clinical manifestations differed greatly between the studies. The calculation of percentage change of CRs size under mTORi treatment was possible only in 14 children. CRs decreased in size in all of these patients. The earliest reported CRs size reduction of at least 30% was after 11 days of everolimus and 5 days of sirolimus therapy. The earliest reported reduction of more than 50% was achieved after 22 and 24 days of everolimus and sirolimus therapy, respectively. Overall, more than 50% reduction in CRs size was achieved in 10 out of 14 patients (71%). For remained 25 out 27 patients (92.6%) for whom detailed data of CRs size was not available, there was information about “significant or remarkable reduction/decrease” or “tumor resolution”. Overall, CRs size redution was reported in 39 out of 41 children (95.1%). In two children there were no data considering the change of CRs size. The increase in CRs size under mTORi treatment was not reported.

In 7 studies, authors reported more or less precise size of CR in more than one time point [24,26,36,43,46,47,52]. The reliable calculation of the correlation between treatment duration and CR reduction was impossible due to data reporting discrepancy and the risk of bias. However, in those seven studies, CR progressively decreased in size with treatment duration.

#### 3.4.3. Effect of mTORi on Clinical Symptoms of CRs

In most patients, mTORi were introduced due to symptomatic CRs (33 in 41, 80.5%), including four children with coexisted SEGA. Symptomatic CRs manifested mostly as cardiac outflow obstruction often with heart failure (27/33, 81.8%) and/or cardiac arrhythmia (13/33, 39.4%) (Table 1 and Table 2). In eight patients (24.2%) there was a duct dependent heart disease resulting from CR (in seven patients) or additional heart defect (one patient). In other patients, mTORi were introduced due to asymptomatic but large CRs (two children), SEGA with asymptomatic CRs (five children), and drug-resistant seizures with asymptomatic CRs (one child).

The therapy with mTORi resulted in a significant clinical improvement or resolution of cardiological symptoms in 30 out of 33 children with symptomatic CRs (90.9%) (22 out of 28 children (78.6%) in the everolimus group and eight out of 13 (61.5%) in the sirolimus group). In seven patients with duct-dependent heart disease resulted from CR the withdrawal of prostaglandin infusion was possible. The patient with duct-dependent heart disease caused by a congenital heart defect could have eventually safely undergone the surgery as RV outflow obstruction resolved under mTORi therapy. Eight patients (19.5%) remained cardiologically asymptomatic. For two patients (4.9%), there were no data available considering the effect of mTORi on the clinical symptoms.

The earliest reported clinical improvement was after two days and one week of everolimus and sirolimus therapy, respectively.

Unfortunately, one patient treated with sirolimus died [40]. Since birth, this child required cardiovascular support due to large CRs originating from the left ventricular posterior wall and extending anteriorly around the right ventricle and right atrium. After 48 h of sirolimus treatment, this child developed drug-resistant arrhythmia requiring extracorporeal life support. The child was considered for heart transplantation but it was declined by the parents. Despite the intensive medical care, the infant developed fulminant drug-resistant sepsis and was reoriented to palliative care, and died.

#### 3.4.4. CRs Size and Clinical Manifestations after mTORi Discontinuation

In only 15 (26.6%) children the duration of the follow-up after mTORi discontinuation was reported. In these patients, the median follow-up period was 7 months (mean 8 months, range 0.5–18 months). Two patients (4.9%) were still treated with everolimus.

Data considering CRs size after mTORi cessation were available for 12 children (29.3%). CRs regrowth was reported in seven patients (7/41, 17,1%) (out out of 28, 14.3%) in the everolimus group and three (three out of 13, 23.1%) in the sirolimus group). Nevertheless, the size of re-grown tumors was still smaller in comparison with the initial size. In 3 children (3/41, 7.3%) mTORi was reintroduced. In five children (12.2%), CRs size after mTORi discontinuation was stable (four (14.3%) in the everolimus group and one (7.7%) in the sirolimus group). Two children (4.9%) had been still treated with everolimus.

Data considering CRs clinical manifestations after mTORi discontinuation were available for 20 children (53.7%). Improvement and complete resolution of the clinical symptoms preserved in 10 patients (10/20, 50%)—7 (seven out of 28, 25%) in the everolimus group and 3 (23.1%) in the sirolimus group. Seven children (seven out of 20, 35%) remained cardiologically asymptomatic. One patient in the everolimus group still experienced some symptoms, but they were milder and there was no left ventricular obstruction that was the reason for mTORi introduction [51]. In one patient, withdrawal of mTORi resulted in the recurrence of a potentially life-threatening arrhythmia that was controlled by reintroduction of everolimus [42].

#### 3.4.5. Safety Profile of mTORi

The safety of mTORi therapy and the occurrence of adverse events were monitored in 33 patients (80.5%). The summary of reported side effects is presented in Table 3. Overall, 34 adverse events were reported. The most common side effects were dyslipidemia (mostly hypertriglyceridemia), recurrent infections, and transient lymphopenia (Table 3). In seven patients (seven out of 41, 17.1%) no side effects were observed during mTORi therapy. For nine patients (9/41, 22%), there were no data considering side effects.

Most of the adverse effects were reported as mild. However, one neonate experienced a pulmonary hemorrhage on the third day of everolimus therapy that was probably associated with highly exceeded everolimus serum concentration [43]. One patient died during the treatment, however, the child was in a critical condition since birth [40]. Only in two studies were the adverse events assessed using the standardized scale, i.e., Common Terminology Criteria of Adverse Events (CTCAE) [42]. CTCAE assesses adverse events in a 5-grade scale in which the 1st grade is the mildest side effect (mild symptoms or asymptomatic) [54]. In these studies, there were only grade 1 and 2 adverse events.

Some authors ceased mTORi treatment when adverse events occurred, other implemented treatment, e.g., omega-3 acids for hypertriglyceridemia or prophylactic treatment with antifungal drugs or antibiotics (Table 1 and Table 2).

#### 3.4.6. Meta-Analysis and Comparison between Everolimus and Sirolimus, and Non-Intervention

Due to the high risk of bias and low quality of data (small studies, significant differences in data reporting within particular studies, and lack of control groups), the meta-analysis or comparison between sirolimus and everolimus were not performed.

Only one study of Aw et al. compared the time of CRs size change under everolimus treatment in four patients with a historical group of 10 children that was not treated with mTORi [24]. Initial CRs size and patients’ age were comparable in both groups. The analysis showed 11.8 times faster CRs size reduction in the group treated with everolimus compared with the control group. CRs size reduction of at least 50% was documented in “everolimus group” at 1.13 ± 0.33 months old, median 29.5 (range 20–40) compared to 72.9 ± 53.03 months in controls (*p* = 0.026) [24].

## 4. Discussion

This review aimed to summarize current data regarding the effectiveness and safety of mTORi in the treatment of CRs in children with TSC. The assumption of the efficacy of mTORi in this indication may be reasonable considering the dysregulation of mTOR pathway in CRs in patients with TSC and reported efficacy of mTORi in the treatment of other tumorous TSC manifestations [12,13,14,15,16,17,18,20].

Almost studies all included in this review reported significant CRs size reduction and clinical improvement under mTORi therapy both in the everolimus and sirolimus group. However, those significantly positive results should be considered with caution as the vast majority of the reports were case studies without a control group and there were no randomized or large cohort studies. Most of CRs in TSC have a natural tendency to regress with age [9]. Therefore, the reduction of CRs size may have resulted from the natural clinical course. However, as the earliest at least 30% and 50% reduction was reported relatively quickly (after five and 22 days, respectively), the acceleration of CRs size reduction due to mTORi is reasonable. Additionally, it needs to be underlined that mTORi were introduced as a life-saving treatment in most cases as ventricular outflow tract obstruction often with heart failure or duct-dependent heart disease connected with a high risk of surgery were the main indications for the therapy. Therefore, clinical improvement, resulting in, e.g., withdrawal of prostaglandin infusion, seems to be a better efficacy indicator than CR size and it was achieved in the majority of patients. One of the included studies compared the rate of CRs size reduction between a small group of patients treated with mTORi and a historical control group [24]. It showed 11.8 times faster CRs reduction under everolimus therapy [24]. Nevertheless, considering the retrospective character of the analysis and small study groups, the scale of that acceleration should be taken with caution. Certainly, large, randomized studies are required to provide high-quality evidence supporting the effectiveness of mTORi in the therapy of CRs in patients with TSC. Soon, the upcoming ORACLE trial may fulfill this gap. In 2020, Stelmaszewski et al. published the protocol of phase II prospective, randomized, placebo-controlled, double-blind, multicenter trial of everolimus as a specific therapy for symptomatic CRs in patients with TSC (ORACLE trial) [23]. The study group will consist of children with TSC and symptomatic CRs that do not need immediate surgery (overall 40 children, 20 patients per arm). The primary trial’s aim is at least a 50% reduction in tumor size on everolimus therapy compared to the placebo arm. Treatment is planned for three months with follow-up until 12 months. Starting dose is set as 4.5 ng/m^2^ with a targeted blood level of 5–15 ng/mL.

As we showed, despite the current lack of high-quality data from randomized or large cohort studies, mTORi have been already used in the treatment of CRs in patients with TSC, especially for symptomatic or large CRs often as an alternative for surgical treatment. However, the optimal duration of mTORi treatment is still unknown. In our review, the reliable calculation of the correlation between treatment duration and CRs reduction was impossible due to data reporting discrepancy. Nevertheless, data included in seven studies may suggest progressive CR reduction with treatment duration [24,26,36,43,46,47,52]. One of the disadvantages of mTORi in comparison to surgery may be the tendency of CRs to regrow after mTORi discontinuation. Only some of the included studies provided the information about the follow-up period after mTORi discontinuation (12 patients). Among them, in seven children (seven out of 12, 58,3%) CRs regrew, however, the tumor was smaller compared to the initial size. Moreover, in most patients, for whom the data were available, a significant clinical improvement persisted after mTORi discontinuation despite tumor regrowth. Additionally, considering the natural tendency of CRs to regress with age, the regrowth of CRs may not be a clinically significant concern. Considering those factors, mTORi may be considered as a therapeutic option for at least temporary treatment of CRs during the symptomatic period. Therefore, the treatment duration should be individually adjusted based on the clinical manifestations and CRs size in follow-up echocardiography. Nevertheless, it is important to underline that until now both everolimus and sirolimus are not registered for the treatment of CRs in patients with TSC. Therefore, it is off-label therapy and bioethics committee acceptance should be obtained before the beginning of treatment.

Everolimus has been more widely tested in TSC in randomized trials (EXIST-1, EXIST-2 and EXIST-3 studies). Unlike sirolimus, it is approved for therapy of some of the TSC manifestations in the US and Europe (SEGA, AML, epilepsy) [55,56]. However, the efficacy of sirolimus in TSC therapy has been also shown in multiple studies [15,16,18,19,57]. The disadvantage of everolimus in comparison to sirolimus is the lack of oral solution. Therefore, tablets of everolimus need to be crushed and dissolved when administered in young children. On the other hand, everolimus in comparison with sirolimus has a greater oral bioavailability and steady-state drug levels after initiation are faster obtained [57]. Currently, studies comparing directly those two drugs in TSC therapy are lacking, although both drugs have been successfully used in the therapy of different TSC manifestations. In this review, due to significant differences in data reporting, we did not perform the comparative analysis between everolimus and sirolimus because of the high risk of bias. In decision making, which drug use for CRs treatment, also other factors including drug form, availability, and reimbursement in particular countries should be taken into consideration.

The results of our review showed the lack of standardization of the dose of mTORi used in children with CRs as the initial dose significantly differed between studies. Due to significant data discrepancy, based on the included studies it is impossible to recommend the initial dose of everolimus or sirolimus for the treatment of CRs in patients with TSC. Some authors applied the doses of everolimus corresponding with EXIST studies (4.5 mg/m^2^/week (about 0.1 mg/day) that seems to be a reasonable strategy. Nevertheless, one should be kept in mind for both everolimus and sirolimus therapy. mTORi are metabolized in the liver by cytochrome P-450 enzymes, mostly CYP3A4 enzyme [57]. CRs are the earliest TSC sign, hence, mTORi were introduced mostly in neonates and infants. The pharmacokinetics of mTORi changes with age. Due to lower activity of liver enzymes (the activity of CYP3A4 is about 30–40% of adult’s activity after 1 month of life) and lower drug clearances, neonates and young infants may be more predisposed to experience side effects as the therapeutic drug concentration may be easier exceeded [58,59]. In included studies, in some neonates, blood concentration of both everolimus and sirolimus were highly above the targeted level, even up to several dozen of ng/mL (Table 1 and Table 2). One neonate experienced pulmonary hemorrhage on the 3rd day of everolimus therapy and the drug level tested one day later was 76.1 ng/mL (targeted level was 5–15 ng/mL) [43]. Therefore, it seems to be reasonable to start treatment in neonates with a lower dose and slowly titrate it to the targeted blood level. More frequent testing of drug serum concentration in that age group may be also considered.

The metabolism of mTORi by cytochrome P-450 enzymes, mostly CYP3A4, may also cause another complication as possible interactions, with drugs influencing the activity of these enzymes such as erythromycin or some antiseizure medications, e.g., carbamazepine. It is important to check if interactions occur and to adjust the dose of mTORi if necessary.

Therapy with mTORi in infants may also influence the vaccination schedule. Due to the immunosuppressive properties of mTORi, live vaccines should be avoided during the treatment. It is recommended to cease treatment for 2 weeks before and 2 weeks after vaccination with the live vaccine [60]. Inactive vaccines can be administered without any changes in mTORi therapy.

mTORi also impair wound healing. It may be an important limitation in children in whom cardiological surgery is planned. It is recommended to temporarily stop the drug administration 7–14 days before the major invasive surgeries. The drug may be re-introduced after the surgical wound is completely healed [60]. Similarly, mTORi should be stopped in patients with major injuries and reintroduced after the injury is healed [60].

Previous studies, mostly large randomized EXIST studies, proved the acceptable safety profile of mTORi in children with TSC [12,13,14,61]. The most frequent side effects are mild and can be easily managed [12,14,60,61]. In most cases, the severity of side effects is dose-dependent [60]. In EXIST studies the incidence of side effects also tended to decrease with the time of therapy [62]. In the literature, the most often side effects include stomatitis, dyslipidemia, infections, diarrhea, and bone marrow suppression. Some side effects may require at least temporary interruption of the treatment [60]. Therefore, treatment with mTORi requires increased awareness and regular laboratory studies e.g., lipid profile, completed blood count, and serum drug concentration. The adverse effects observed in the studies included in our review were mostly mild and corresponded with those reported in the literature (Table 3). However, only two authors assessed the severity of adverse events using an objective tool as a standardized CTCAE scale.

As the reports included in our analysis had focused on the cardiological manifestations, in most studies, there was no information about the influence of mTORi on other TSC manifestations, including seizures. Considering the role of overactivity of the mTOR pathway in the pathogenesis of TSC, early implementation of mTORi in neonates and infants with TSC may be also explored in the future as a possibility to prevent some of the TSC manifestations. In 2013 Kotulska et al. reported a unique case report of two monozygotic twins with TSC and SEGA [63]. Only one of the sisters qualified to the EXIST trial and she was treated with everolimus. After 27 months, not only SEGA volume had been reduced, but she also did not have renal AML and facial angiofibromas that were observed in her twin sister [63].

### Limitations of the Study

Limitation on the search and selection level: the search of articles and the first phase of the selection were performed only by one reviewer. Therefore, the risk of bias might be higher in comparison with the search performed independently by at least two reviewers.

We included only reports of children with confirmed TSC diagnosis. However, because multiple CRs are highly associated with TSC and most of the clinical manifestations of TSC develop with time some of the excluded articles may have also reported children who were later diagnosed with TSC.

An additional limitation is the fact that we included only articles published in English or Polish and we searched four databases. Therefore, some articles might have been omitted.

Limitation at the outcome level: The most important limitation of included studies is a high risk of bias due to the lack of randomization and control group in the majority of studies. Considering the fact that all of the included studies were case reports or case series there is also the risk bias in the reported positive effect of mTORi on the CRs size and clinical manifestations as case reports in which the particular treatment was not effective are less commonly reported and published.

An additional limitation is the fact that CRs in TSC spontaneously regress with time in most children. Therefore, the effect of mTORi on CRs size is difficult to be unequivocally assessed in studies without a control group.

The additional limitation at the outcome level are the significant differences in data reporting and its quality between the studies.

Limitations of presented systemic review: The main limitation of this systematic review is the lack of high-quality data from randomized or large cohort studies. Therefore, metanalysis and comparison between sirolimus and everolimus were not performed.

## 5. Conclusions

In recent years, mTORi have been more and more widely used in the treatment of various TSC manifestations. Although there is evidence for the effectiveness and safety of mTORi in the treatment of CRs, currently, due to the lack of high-quality studies, the evidence is not sufficiently robust to unequivocally recommend this therapy in every patient. However, based on the available data and considering the tendency to spontaneous regression of CRs in most patients, mTORi may be considered as a temporary therapeutic option for symptomatic CRs in children with TSC, especially when the risk of surgical intervention is significant. Due to the immaturity of liver enzymes, it is important to slowly introduce mTORi and frequently check the drug serum concentration in neonates and young infants. The upcoming randomized trial (ORACLE) may provide more reliable, evidence-based results on the effectiveness of mTORi in the treatment of CRs among children with TSC.

## Figures and Tables

**Table 1:** Characteristics of children treated with everolimus.

Author, Year	Localization of the Largest or Symptomatic CR	initial Size of the Largest or Symptomatic CR	CR Clinical Symptoms/Reason to Start mTORi	Age at mTORi Introduction	Treatment Duration	Initial mTORi Dose	Follow-Up of mTORi Serum Level	Effect of mTORi on CRs Volume	Effect of mTORi on Clinical Symptoms	Follow-Up Period after mTORi Withdrawal	CRs Volume at the End of Follow-Up	CR Clinical Manifestations at the End of Follow-Up
Aw et al., 2017 [24]; Goyer et al., 2015 [53]; Mlczoch et al., 2015 [34]												
1	LV	8 × 7 mm	significant pressure gradient in the LV outflow tract	1 day	36 days	0.1 mg/day (4.5 mg/m^2^/week), targeted level 5–15 ng/mL	after 10 days; drug level—10.2 ng/mL	After 36 days; 50% reduction of the largest CR, other 3 disappeared	no cardiological symptoms	at least 18 months	progressive increase in CR size: on day 112: CR size was 3.2 × 2.7 mm on day 576: CR size was 6.6 × 4.1 mm	no clinical symptoms
2	LV	15 × 9 mm	no clinical symptoms, multiple tumors occupying LV and concern about possible outflow tract obstruction	4 days	46 day	0.1 mg/day (4.5 mg/m^2^/week); targeted level 5–15 ng/mL	after 6 days; drug level—11 ng/mL	After 22 days; 50% reduction of CR size	remained asymptomatic	at least 7 months	progressive increase in CR size On the 48th day of life—12.4 × 7.8 mm On the 268th day of life–17.5 × 13.2 mm	remained asymptomatic
3	RV	11 × 6 mm	SEGA, CR were asymptomatic	9 days	no data	0.1 mg/day (4.5 mg/m^2^/week); targeted level 5–15 ng/mL	after 13 days; drug level −5.4 ng/mL	After 11 days; CR size was 6.5 × 3.1 mm (30–50% reduction) After 1 month; CR was undetectable	remained asymptomatic	still treated with everolimus	-	-
4	RV	no data	duct dependent RVOT obstruction, heart failure	21 days	planned for 1 year	3 mg/m^2^; targeted level of 4–5 ng/mL	no data	after 28 days; CR regression (no detailed data)	after 3 months; a significant improvement, no obstruction in RVOT	2 weeks	CR dramatically increased in size (everolimus was re-introduced)	do data
Bornaun et al., 2016 [25]	LV, RV	1.3 cm^2^	cyanosis, hypotonia, LV hypertrophy, obstruction of LVOT, mild obstruction of RVOT, inoperable CR	7 days	overall 6 months	2 × 0.25 mg twice a week; targeted level 2.6–6.1 ng/mL	drug level varied from 0.4 to 2.6 ng/mL	(1) after 28 days; significant reduction of CR (everolimus was ceased) (2) after 180 days of everolimus reintroduction; marked decrease of CR size	after 4 weeks; resolution of LV outflow tract obstruction	(1) 10 days (after 1st drug withdrawal) (2) 12 months (after 2nd drug withdrawal)	(1) significant regrowth of all CR (2) stable CR size	no data
Castro-Monsalve et al., 2018 [47]	IVS	no data	cardiac arrest, severe hemodynamic instability, cardiac failure	neonate	no data	0.1 mg/day	drug level between 5–8 ng/mL	After 31 days; 60% reduction of CR size After 60 days; resolution of CR	no data	no data	no data	no data
Choudhry et al., 2015 [48]	RV, LV	from 3 to 12 mm	SEGA; CR were asymptomatic	neonate	no data	no data	no data	after 30 days; the apparent resolution of all CR (no detailed data)	remained asymptomatic	no data	no data	remained asymptomatic
Colaneri et al., 2016 [49]	LV	40 × 35 × 40 mm (transverse diameter in thorax MRI—35 × 25 mm)	severe reduction of LV volume resulting in duct dependent heart disease, sporadic ventricular extrasystoles, large renal angiomyolipoma; surgery was contraindicated	7 days	10 weeks	0.25 mg/day (0.11 mg/kg; 1.5 mg/m^2^), targeted level 5–15 ng/mL	after 5 days; drug level—9.1 ng/mL;	After 70 days; CR size—23 × 9 mm in thorax MRI (30–50 % reduction)	after 10 days; extrasystoles disappeared after 3 weeks; normal ventricular function	9 months	Stable	no clinical symptoms
Demir et al., 2012 [50];	RV, IVS, LV	CR sizes ranged from 5 to 25 mm	cyanosis, RV heart failure, obstruction of RV inflow, CR ineligible for surgery	neonate	2.5 months	0.25 mg every 6 h 2 days per week; targeted level 5–15 ng/mL	after 4 doses drug level was 83.5 ng/mL	After 70–75 days; CR remarkably decreased in size (no detailed data)	after 2.5 months; hemodynamic instability improved	2 months	no data	no clinical symptoms
Dogan et al., 2015 [51]	LV	24 × 21 mm and 22 × 20 mm	cyanosis, LV obstruction, inoperable tumors	neonate	3 months	0.25 mg two times per day, 2 days per week; targeted level of 5–15 ng/mL	drug level ranged from 3.6 to 7.8 ng/mL	After 60 days; significant CR reduction	After 2 months; no hemodynamic instability, relief of LV obstruction	15 months	no data	severe mitral insufficiency, moderate LV dilatation, WPW in ECG
Garg et al., 2018 [52]	RV	40 × 37 × 30 mm	ventricular tachycardia, hypotension, mild/moderate tricuspid insufficiency; high risk of surgery	neonate (around 4–5th day of life)	no data	0.08 mg/day (0.3 mg/m^2^/day)	no data	After 14 days; a slight decrease of CR size After 30 days rapid involution of the CR	Significant clinical improvement after a few days of treatment	still treated, but the dose was not weight-adjusted	-	-
Hoshal et, 2015 [26]	LV, intrapericardial tumor extending along the aortic root	no data	circulatory collapse, RVOT obstruction, cardiac enlargement, disqualification from the surgery	neonate or infant	more than 10 months	0.5 mg/day	no data	After 60 days; regression of CR After 300 days; CR almost disappeared	after 2 months; improvement after 10 months; normal LV ejection fraction	no data	no data	no data
Kim et al., 2019 [27]	no data	45 mm	hemodynamically unstable arrhythmia and SEGA	older than 15 months	no data	no data	no data	After 150 days; complete regression of CR	no data	no data	no data	no data
Martínez-García et al., 2018 [33]	LV	47 × 40 mm	Giant CR occupying almost whole LV resulting in duct dependent heart disease, cardiomegaly, incomplete left bundle branch block with severe repolarization disorder	36 days	no data	0.25 mg two times per day only 2 days a week	no data	After 90 days; CR size 22 × 29 mm (30–50% reduction)	after 3 months; normal ejection fraction	no data	no data	no data
Mohamed et al. 2014 [35]	IVS, RV	16 × 11 mm	RVOT obstruction with heart failure; high risk of surgery, additional duct dependent heart defect	20 days	34 days	0.1 mg/day (about 4.5 mg/m^2^/week) targeted level of 5–15 ng/mL	after 11 days; drug level 11 ng/mL	After 34 days; significant reduction of CR	after 34 days; no obstruction of RV outflow tract (the child underwent surgical intervention on the 88th day of life due to a structural heart defect)	12 months	stable	no clinical symptoms
Öztunç et al., 2015 [38]	LV, RV, IVS	no data	pharmacoresistant supraventricular tachycardia	neonate	4 weeks	0.25 mg 2 times per day twice a week	no data	After 15 days; CR started to shrink	after 8 days; the frequency and duration of tachycardia diminished	6 months	stable	no clinical symptoms
Prasad et al. 2020 [41]	LV	31 × 41 mm	congestive heart failure, respiratory dysfunction, LV dysfunction	neonate	16 weeks	4.5 mg/kg/m^2^ weekly	no data	After 70 days; CR size was 9 × 11 mm (>50% reduction)	improvement of respiratory function	no data	no data	no clinical symptoms
Saffari et al., 2016 [42] (study reported 8 patients with CR treated with mTORi)	no data	no data	(6 children) symptomatic CR—obstruction of cardiac outflow or arrhythmia; (1 child) SEGA and symptomatic CR; (1 child) SEGA and asymptomatic CR	median age 10.5 days (2 days—5 months); neonates—5 children; infants— 3 children	no data	Infants up to 3 months—doses ranging from 0.05–0.3 mg; infants > 5 months—doses ranging from 1 to 5 mg/day	In 1 patient toxic drug level of around 100 ng/mL after initial dose of 0.4–0.45 mg (1.5–2 mg/m^2^)	In all patients CR decreased in size	clinical improvement	no data	no data	In one patient recurrence of potentially life-threatening arrhythmia after everolimus cessation. The reintroduction of the drug controlled the arrhythmia
Shibata et al., 2019 [43]	RV	35 × 21 mm	duct dependent obstruction of the LVOT	4 days	less than 35 days	0.2 mg/kg/day; targeted level 5–15 ng/mL	on 4th day; drug level: 76.1 ng/mL, drug was transiently withdrawn	After 16 days; CR size—28 × 15 mm (<30% reduction) After 38 days; CR size—24 × 11 mm (30–50% reduction)	after 4 days; resolution of duct dependent heart disease	no data	no data	no data
Tibero et al., 2011 [44]	LV	no detailed data	SEGA, CR were asymptomatic	5 years	13 months	no data	drug level between 2.3 and 7.1 ng/mL	After13 months; near-resolution of CR	remained asymptomatic	no data	no data	remained asymptomatic
Wagner et al., 2015 [45]	LV	21 × 37 × 21 mm	LVOT obstruction (partially duct dependent)	2 days	19 days	1.5–2 mg/m^2^; targeted level 5–15 ng/mL	after 4 days; drug level of 108 ng/mL; the drug was stopped for 4 days	After 21 days; CR size—10 × 28 × 13 mm (30–50% reduction)	improvement—prostaglandin infusion was ceased after 2 days of therapy	5 months	stable	no clinical symptoms

Abbreviations: CR—cardiac rhabdomyoma(s), IVS—intraventricular septum, LV—left ventricle, LVOT—left ventricle outflow tract, mTORi—mTOR inhibitor, RA—right atrium, RV—right ventricle, RVOT—right ventricle outflow tract.

**Table 2:** Characteristics of children treated with sirolimus.

Author, Year	Localization of the largest or Symptomatic CR	Initial Size of the Largest or Symptomatic CR	CR Clinical Symptoms/Reason to Start mTORi	Age at mTORi Introduction	Treatment Duration	Initial mTORi Dose	Follow-Up of mTORi Serum Level	Effect of mTORi on CR Volume	Effect of mTORi on Clinical Symptoms	Follow-Up Period after mTORi Withdrawal	CR Volume at the End of Follow-Up	CR Clinical Manifestations at the End of Follow-Up
Breathnach et al., 2014 [36]	LV	15 × 12 mm	obstruction of LVOT resulting in temporary duct dependent heart disease; high risk of surgery	10 days	24 days	0.5 mg/once daily; targeted level 20 ng/mL	After 7 days; drug level—26 ng/mL	After 5 days; CR size—7 × 8 mm (30–50% reduction) After 24 days; CR size—5 × 4 mm (>50% reduction)	no cardiological symptoms	7 months	CR slightly increased in size	no clinical symptoms
Knadler J et al., 2020 [28]	RA	(a) 51 mm (b) 8.5 × 8 mm	Duct dependent heart disease due to limited tricuspid valve inflow caused by CR and additional atrial septal defect with right to left shunt	3 days	until at least 6 months of age	targeted drug level 8–12 ng/mL	initially slightly supratherapeutic drug level (no detailed data)	After 90 days; remarkable reduction of CR size	after 3 months; no cardiological symptoms	no data	no data	no data
Lawley et al., 2019 [29]	IVS encroaching on both ventricles	no data	LVOT obstruction, cardiomegaly	3 days	2 months	0.25 mg daily; targeted drug level 5–15 ng/mL	after 8 days; drug level was 69.7 ng/mL (the drug was transiently withdrawn)	After 11 days; significant regression of CR	clinical improvement	(1) 1 month (2) 9 months after sirolimus reintroduction	(1) substantial increase in CR size; (2) sustained reduction of CR size	no data
Lee et al., 2017 [30]	LV	5.2 × 3.6 mm	severe LVOT obstruction, high risk of surgery	18 days	10 weeks	0.25 mg daily	after 14 days; drug level of 42.1 ng/mL;	After 43 days; CR size—2.3 × 1.9 mm (>50% reduction)	after 43 days; no cardiological symptoms	7 months	no data	no clinical symptoms
Lucchesi et al., 2018 [31](the study of 3 consecutive cases)	no data	no data	SEGA in all patients; 1 patient also had a paroxysmal supraventricular tachycardia	less than 12 months—(mean age 7 months)	at least 6 months	1 mg/m^2^/day; targeted level 4–10 ng/L	no data	Median time 1.9 months (57 days) (ranged from 0.8 to 4.7 months) In 2 patients complete regression; In 1 patient more than 50% size reduction	no cardiological symptoms	no data	no data	no data
Mao et al., 2017 [32]	LA and RV	1 × 0.89 mm and 1.8 × 1.7 mm	drug-resistant seizures, CR were asymptomatic	90 days	no data	no data	targeted level of 5–10 ug/l	After 90 days; regression of CR	remained asymptomatic	no data	no data	no data
Ninic et al., 2016 [37]	IVS	no data	pharmacoresistant cardiac arrhythmia, slightly diminished LV contractility	3 years	no data	1 mg/m^2^ twice a day	after 5 days; sirolimus level 6.6 ng/mL (within therapeutic range)	no data	after 14 days; normalization of the heart rhythm	no data	no data	no data
Patel et al., 2018 [39]	LV pericardium	30 × 45 mm	small pericardial effusion after birth, decreased LV systolic function, high risk of surgery	neonate	4 weeks	no data	no data	Significant regression of CR	after 1 week; notable improvement of cardiac function after 4 weeks; LV function normalized	1 month	stable	no clinical symptoms
Prabhu et al., 2018 [40]	originated from LV and extended to RV and RA	no data	respiratory distress, cardiac failure, the patient required ongoing cardiovascular support; ST depression in ECG	neonate	no data	no data	no data	no data	After 48 h, fulminant ventricular ectopy with associated systemic hypotension occured (antiarrhythmic drugs and extracorporeal life support were required)	no data	no data	patient died due to fulminant sepsis and necrotizing enterocolitis
Weiland et al., 2018 [46]												
1	LV, RV—apex (encroaching on the LV and RV)	25 × 25 × 33 mm	no symptoms, but CR was massive—concern of possible impairment of ventricular function; high risk of surgery	neonate	4 weeks	initial dose 0.1 mg/kg daily; targeted level of 5–15 ng/mL	at 4th week drug level—22.5 ng/mL drug was ceased	After 28 days; CR size—9 × 9 × 9 mm (>50% reduction)	remained asymptomatic	9 months	CR size increased to 16 × 12 × 10 mm	remained asymptomatic
2	LV	(a) 22.1 × 14.5 × 8 mm (b) 10.6 × 9.6 × 9.7 mm	Mild obstruction of the LVOT	neonate	no data	initial dose 0.1 mg/kg every 12 h; targeted level of 5–15 ng/mL	at 12th day; drug level—24.3 ng/mL	After 12 days: (a) CR size—16 × 7 × 10 mm (<30% reduction) (b) CR size—8 × 6 × 5 mm (30–50% reduction) After 6 weeks; (a) 10 × 9 × 7 mm (>50% reduction); (b) 6 × 5 × 5 mm (>50% reduction)	after 12 days; improvement—no evidence of outflow tract obstruction	no data	no data	remained asymptomatic

Abbreviations: CR—cardiac rhabdomyoma(s), IVS—intraventricular septum, LV—left ventricle, LVOT—left ventricle outflow tract, mTORi—mTOR inhibitor, RA—right atrium, RV—right ventricle.

**Table 3:** Summary of mTOR inhibitor side effects.

Side Effect	Number of Particular Adverse Events	
	Everolimus	Sirolimus
dyslipidemia (mostly hypertriglicerydemia)	6	3
transient lymphopenia	3	0
infections	3	1 (sepsis)
mouth ulcers/mucositis	2	1
acne	2	0
changes in phosphate levels	2	0
increased cholinesterase	2	0
transient neutropenia	2	1
diarrhea/constipation	1	1
transient hypokalemia	1	0
transient anemia	1	0
pulmonary hemorrhage	1	0
elevated liver enzymes	1	0
decreased CD4/CD8 ratio	1	0
hyponatremia	1 (association with everolimus is doubtful because the patient also received diuretics)	0
fever without evidence of infection	0	1
none reported (no. of patients)	7	4
no data (no. of studies)	7	2

Patients in whom side effects were monitored (*n* = 33). There was a possibility of more than 1 adverse event per patient.
